# The burden of obesity in primary care in Italy: Italian real-world overweight/obesity study (ITROS)

**DOI:** 10.1007/s40519-025-01791-8

**Published:** 2025-10-22

**Authors:** Silvio Buscemi, Luca Busetto, Uberto Pagotto, Paolo Sbraccia, Clara Bagatin, Simona Barzaghi, Valeria Pegoraro, Chiara Gerbino, Dario Delmonte, Laura Clementi

**Affiliations:** 1 Department of Promozione della Salute, Materno-Infantile, Medicina Interna e Specialistica di Eccellenza (PROMISE), University of Palermo, Palermo, Italy; 2 Unit of Clinical Nutrition, Obesity and Metabolic Diseases, University Hospital Policlinico “P. Giaccone”, Edificio 2a - Piazza Delle Cliniche, 2 - 90127 Palermo, Italy; 3 Department of Medicine–DIMED, University of Padua, Padua, Italy; 4 Division of Endocrinology and Diabetes Prevention and Care, IRCCS Azienda Ospedaliero-Universitaria di Bologna, Bologna, Italy; 5 Department of Medical and Surgical Sciences (DIMEC), Alma Mater Studiorum University of Bologna, Bologna, Italy; 6 Department of Systems Medicine, University of Rome Tor Vergata, Rome, Italy; 7 Internal Medicine Unit - Obesity Center, University Hospital Policlinico Tor Vergata, Rome, Italy; 8 IQVIA Solutions Italy Srl, Milan, Italy; 9 Department of Drug Science and Technology, University of Turin, Turin, Italy; 10 Medical Affairs, Boehringer-Ingelheim, Milan, Italy

**Keywords:** Obesity, Severe overweight, General practitioners, Clinical characterization, Healthcare resources utilization

## Abstract

**Purpose:**

Generating real-world evidence on individuals living with severe overweight or obesity in Italy, focusing on their characterization and management from general practitioners (GPs) perspective.

**Methods:**

This was a non-interventional longitudinal observational cohort study using data from the Italian IQVIA Longitudinal Patient Database (LPD), conducted in collaboration with a working group from the ‘Società Italiana di Obesità’. The study included individuals with body mass index (BMI) ≥ 27 kg/m^2^ during ‘January 2018–June 2022’. Data on clinical conditions, GP interventions (including drug prescriptions, and referrals for laboratory tests, instrumental examinations, and specialist visits), and hospitalizations were collected during the year preceding (baseline) and following BMI recording. Data were analyzed according to time (follow-up versus baseline) and BMI thresholds.

**Results:**

The final cohort consisted of 134,776 individuals: 44.9% with severe overweight, 36.7% with class I, 12.9% with class II, and 5.6% with class III obesity. Overall mean age was 59.9 years and men accounted for 52.9%. Mean age and male proportions decreased across increasing BMI categories. Most frequently recorded conditions during follow-up were hypertension (51.4%), cardiovascular disease (27.5%), and type-2 diabetes (25.1%). Proportions of subjects presenting with clinical conditions and of individuals requiring clinical interventions were higher during follow-up compared to baseline. The likelihood of presenting with most of clinical conditions and interventions increased with BMI.

**Conclusion:**

Patients living with overweight or obesity experience a significant worsening of their health status which increases healthcare resources utilization. Public health interventions could benefit from supporting GPs with training and resources to enhance obesity management and improve patient outcomes.

*Level of evidence*: Level III: Evidence obtained from well-designed cohort or case–control analytic studies

**Supplementary Information:**

The online version contains supplementary material available at 10.1007/s40519-025-01791-8.

## Introduction

Obesity is a complex disease characterized by an excessive or abnormal accumulation of fat, which significantly increases the risk of acute events and chronic diseases [1]. Indeed, obesity is associated with an augmented risk for multiple complications including type-2 diabetes (T2D), metabolic dysfunction-associated steatotic liver disease (MASLD), cardiovascular disease (CVD), and many mechanical complications such as osteoarthritis and obstructive sleep apnea (OSA) [2]. Obesity management might rely on lifestyle interventions like diet, exercise, and behavioral changes, which; however, have a modest impact on weight [3], and on therapeutical interventions, including bariatric surgery and pharmacological treatments. Bariatric surgery can lead to significant weight loss, but is not scalable at population level [4]. The latest approved pharmacological treatments for obesity management in Italy are semaglutide, a glucagon-like peptide-1 receptor agonists (GLP-1 RA) [5], and tirzepatide, which is part of a new therapeutic class that is a combination of glucose-dependent insulinotropic polypeptide and GLP-1 RA [6]. A large pipeline of entero-pancreatic hormone-based pharmacotherapies is under development, aiming to enhance/complement the efficacy and mechanisms of action of GLP-1 RAs.

Over the past five decades, global prevalence of obesity has been steadily increasing, leading to the declaration of an “obesity pandemic” [1]: recent estimates from the Global Burden of Disease initiative indicate that approximately one-third of the global population is affected by overweight or obesity [1]. In Italy, data from the Surveillance Program Progressi delle Aziende Sanitarie per la Salute [7] and the latest Obesity Monitor [8] consistently revealed that in 2023 about 17 million and 6 million adults were affected by overweight and obesity, respectively. This poses significant challenges to the financial sustainability of the Italian National Health System (Servizio Sanitario Nazionale, SSN) and a substantial economic burden on society [9]. The Italian Government has implemented various population- and individual-based interventions aimed at curbing the increasing trend of obesity [1]. However, recent reports indicate that people living with overweight and obesity has increased by 14% and 38% respectively in the last 20 years [8], and projections suggest that prevalence of obesity could double its 2000 level in 2030 reaching 13% [10].

Primary care is the most suitable setting to assess the health status of the Italian population—being less susceptible to selection bias [4]; among healthcare professionals, General Practitioners (GPs) serve as the frontline providers in primary care, and are expected to play a pivotal role in the initial assessment, treatment, and screening of adults living with obesity [11]. For these reasons, and in light of poor availability of real-world Italian data that might instead contribute to tailor interventions on actual needs improving campaigns effectiveness, authors developed the Italian Real-world overweight/Obesity Study (ITROS) to generate evidence on people living with severe overweight or obesity. Specifically, ITROS focused on characterizing affected individuals and evaluating the management of the condition and its complications over time from GP perspective.

## Methods

This was a noninterventional, longitudinal, observational cohort study using secondary data conducted in collaboration with a Working Group from the Società Italiana Obesità (SIO), that revised the study protocol and results, and provided data interpretation.

### Data source

Italian IQVIA Longitudinal Patient Database (LPD) is a computerized network of GPs who contribute to a centralized database reflecting the normal clinical practice of a national sample of independent GPs and has been extensively used in real-world evidence studies [12]. It enables longitudinal analysis of data from records related to prescription practices and healthcare resources utilization (HRUs) in daily clinical practice. Drugs’ prescriptions and medical diagnoses are recorded by GPs and comply with the World Health Organization (WHO) Anatomical Therapeutic Chemical (ATC) classification system, and the International Classification of Diseases, 9th revision (ICD-9), respectively. Currently, ~ 900 GPs contribute to the database, providing data of ~ 1.2 million people. IQVIA LPD represents the general Italian population in terms of demographic characteristics being a reliable source of information [13]. The geographic distribution of both GPs and patient samples reflects that of the Italian resident population [14].

### Study design and population

A selection period (01-Jan-2018 to 30-Jun-2022) was considered to identify people with at least one measurement of BMI ≥ 27 kg/m^2^, i.e., people eligible for the analysis. For each participant, the date of first BMI record ≥ 27 kg/m^2^ during the selection period was defined as Index Date. Individuals were observed during the 12-month period preceding Index Date (baseline), and the 12-month period starting at Index Date (follow-up). Patients meeting any of the following criteria were excluded from the analysis: (1) no data availability for the entire period, (2) age at Index Date < 18 years, (3) pregnancy status during the entire period, (4) cancer diagnosis during baseline, (5) diagnosis of amputation during the entire period, (6) evidence of bariatric surgery before baseline. The BMI cut-off of 27 kg/m^2^ was chosen in light of latest obesity pharmacological treatment indications [5, 6]. Once included in the final cohort, individuals were classified according to their Index Date BMI as subjects with severe overweight (OW) (27 kg/m^2^ ≤ BMI < 30 kg/m^2^), subjects with class I obesity (OB-I) (30 kg/m^2^ ≤ BMI < 35 kg/m^2^), subjects with class II obesity (OB-II) (35 kg/m^2^ ≤ BMI < 40 kg/m^2^), or subjects with class III obesity (OB-III) (BMI ≥ 40 kg/m^2^). Information on age and sex was collected at Index Date. The presence of clinical conditions of interest, information on GPs interventions aimed to manage severe overweight/obesity and its complications (which included drug prescriptions, referrals for laboratory tests, instrumental examinations, specialist visits and bariatric surgery), and information on hospitalizations were evaluated both during baseline and follow-up.

### Statistical analysis

All the information was descriptively analyzed for the overall cohort and with a stratification according to BMI group, separately for baseline and follow-up, and reported as frequencies and percentages. Age and sex-adjusted multivariate logistic models were run to estimate the likelihood of presenting each clinical condition and intervention being considered during follow-up according to BMI with severe OW group as reference category. Results from the models were reported as Odds Ratios (ORs) with 95% confidence intervals (CIs). *P* values < 0.05 were considered as statistically significant. The analyses were conducted using SAS^®^ Enterprise Guide^®^ software.

## Results

**Fig. 1:**
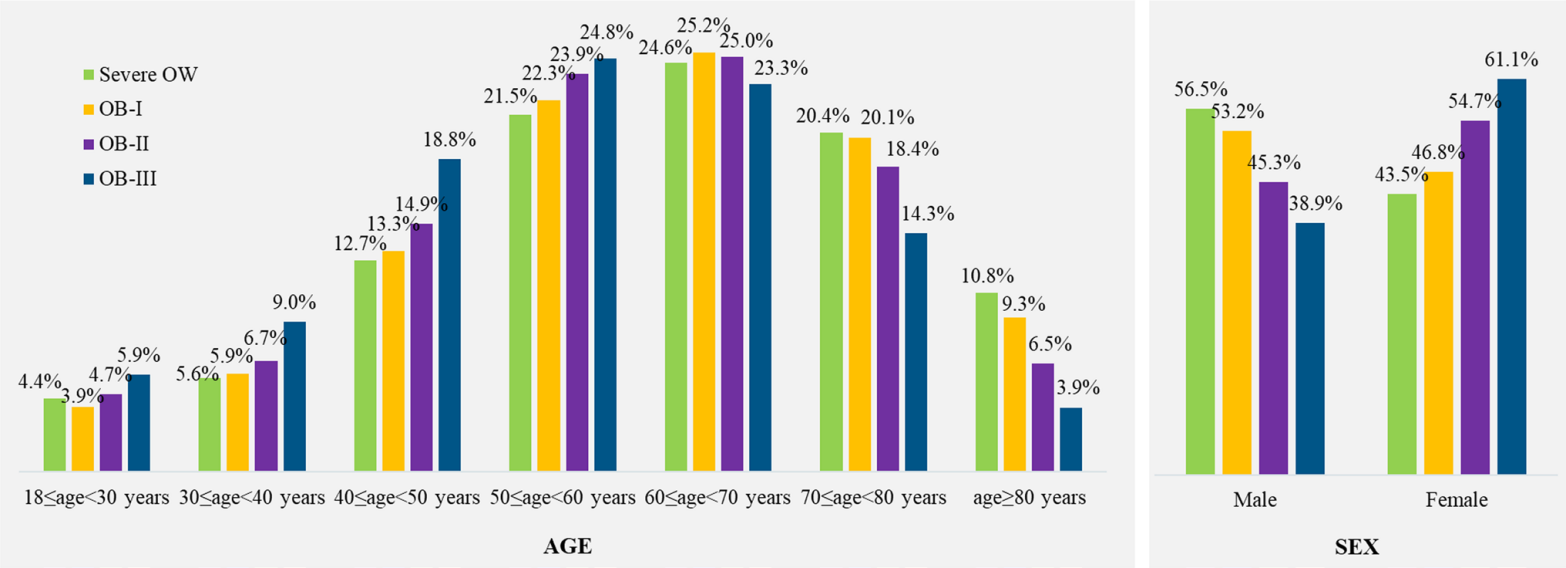
Age and sex according to body mass index (BMI) group. OW overweight, *OB-I* obesity class I, *OB-II* obesity class II, *OB-III* obesity class III

177,505 people with BMI ≥ 27 kg/m^2^ were identified during the selection period. After applying the exclusion criteria, the final cohort consisted of 134,776 individuals: 60,468 (44.9%) with severe OW, 49,486 (36.7%) with OB-I, 17,330 (12.9%) with OB-II, and 7492 (5.6%) with OB-III. People with OB-I, II, and III accounted respectively for 67%, 23%, and 10% of people with obesity (Supplementary Information, Fig. 1S). Overall mean age (± standard deviation, SD) was 59.9 (± 15.4) years, with a decreasing trend observed across increasing BMI categories: mean age (± SD) was 60.6 (± 15.7), 60.2 (± 15.2), 58.4 (± 15.0), and 55.3 (± 14.9) years for severe OW, OB-I, OB-II, and OB-III, respectively; higher proportions of individuals aged < 60 years were observed for OB-II and OB-III, while percentages of individuals aged ≥ 70 years were higher for severe OW and OB-I (Fig. 1). Although males represented the majority of the cohort (52.9% vs. 47.1%), the proportion of females increased progressively with higher BMI categories (Fig. 1).

**Fig. 2:**
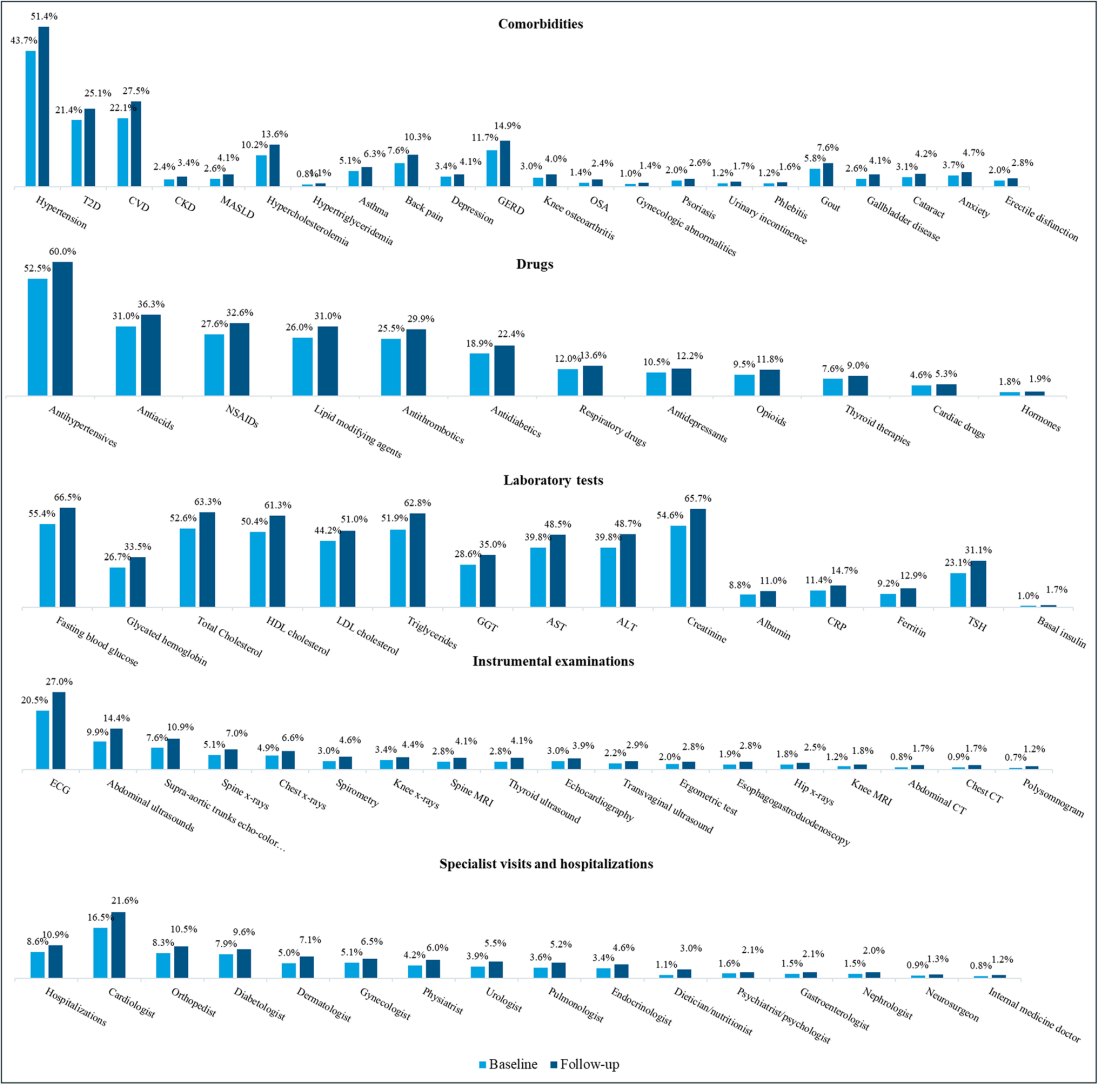
Overall proportions of subjects with each comorbidity, 1 + referral for each drug, laboratory test, instrumental examination, specialist visit, and 1 + hospitalization during baseline and follow-up T2D type-2 diabetes, *CVD* Cardiovascular disease, *CKD* chronic kidney disease, *MASLD* metabolic dysfunction-associated steatotic liver disease, *GERD* gastroesophageal reflux disease, *OSA* obstructive sleep apnea, *NSAIDs* non-steroidal anti-inflammatory drugs, *HDL* high-density lipoprotein, *LDL* low-density lipoprotein, *GGT* gamma-glutamyl transferase, *AST* aspartate aminotransferase, *ALT* alanine aminotransferase, *CRP* C-reactive protein, *TSH* thyroid-stimulating hormone, *ECG* electrocardiogram, *CT* computed tomography, *MRI* magnetic resonance imaging

Subjects with additional BMI measurements during follow-up represented 26.4% of the cohort. No major variations were observed in mean BMI compared to Index Date, except for people with OB-III, who showed a slight decrease from 44.1 (± 4.3) to 42.2 (± 5.0) kg/m^2^. Proportions of subjects in the overall cohort presenting with the different comorbidities, drugs prescriptions, laboratory tests, instrumental examination, and specialist visit referrals, and with evidence of hospitalizations were always higher during follow-up compared to baseline (Fig. 2). Focusing on follow-up, the most frequently recorded condition was hypertension (51.4%), followed by CVD (27.5%), and T2D (25.1%). The highest relative increase in terms of affected people was observed for OSA (+ 71.4%), MASLD (+ 57.7%), and gallbladder disease (+ 57.7%). Among drugs more frequently prescribed we found antihypertensives (60.0%), antiacids (36.3%), non-steroidal anti-inflammatory drugs (32.6%), and lipid modifying agents (31.0%). Conversely, pharmacological therapies indicated for overweight and obesity at the time of study conduct—specifically bupropion/naltrexone, orlistat, and liraglutide—were prescribed to less than 0.5% of the cohort (data not shown). A core set of laboratory tests—including fasting blood glucose, total, low-and high-density lipoprotein cholesterol, triglycerides, and creatinine—was consistently prescribed to ≥ 50% of the cohort during follow-up, while albumin, C-reactive protein, ferritin, and basal insulin tests were prescribed less frequently (< 15%, Fig. 2). Electrocardiogram, abdominal ultrasounds, and supra-aortic echo-color Doppler were the only instrumental examinations requested for ≥ 10% of the cohort (27.0%, 14.4%, and 10.9% respectively). While GPs rarely reported advising patients on diet or physical activity (in 3.0% and 2.6% of the cohort, respectively), they frequently referred patients to a cardiologist (21.6%) and an orthopaedist (10.5%). Frequencies of consultation of the other specialists did not reach 10%. Overall hospitalization rate was 10.9% (Table 2).

For T2D, asthma, depression, OSA, back pain, and knee osteoarthritis the proportion of affected individuals during follow-up increased progressively across BMI groups. The greatest differences were observed for T2D, with proportions of affected individuals ranging from 22.0% among severe OW to 31.2% in the OB-III group, and OSA, with individuals with the condition representing 1.3% of people with severe OW and 6.7% of OB-III group. In general, for the majority of the diseases, higher percentages of affected subjects were observed for OB-II and OB-III (Table 1).

**Table 1:** Proportions of subjects with each clinical condition/disease during follow-up stratified by body mass index (BMI) group

Comorbidity	Severe OW (*N* = 60,468)		OB-I (*N* = 49,486)		OB-II (*N* = 17,330)		OB-III (*N* = 7492)	
	*N*	(%)	*N*	(%)	*N*	(%)	*N*	(%)
CVD	16,602	(27.46)	14,061	(28.41)	4568	(26.36)	1808	(24.13)
Hypertension	28,619	(47.33)	26,590	(53.73)	9795	(56.52)	4199	(56.05)
T2D	13,287	(21.97)	13,147	(26.57)	5012	(28.92)	2336	(31.18)
CKD	1948	(3.22)	1736	(3.51)	620	(3.58)	258	(3.44)
MASLD	2297	(3.80)	2142	(4.33)	793	(4.58)	291	(3.88)
Hypercholesterolemia	8851	(14.63)	6846	(13.83)	2002	(11.6)	662	(8.84)
Hypertriglyceridemia	612	(1.01)	625	(1.3)	184	(1.06)	85	(1.13)
Asthma	3417	(5.65)	3190	(6.45)	1270	(7.33)	663	(8.85)
Back pain	6037	(9.98)	5184	(10.48)	1821	(10.51)	804	(10.73)
Depression	2347	(3.88)	2023	(4.09)	771	(4.45)	372	(4.97)
GERD	8915	(14.74)	7311	(14.77)	2682	(15.48)	1158	(15.46)
Knee osteoarthritis	1859	(3.07)	2177	(4.40)	912	(5.26)	449	(5.99)
OSA	804	(1.33)	1203	(2.43)	726	(4.19)	502	(6.70)
Gynecologic abnormalities	348	(1.32)	329	(1.42)	135	(1.42)	92	(2.01)
Psoriasis	1496	(2.47)	1301	(2.63)	499	(2.88)	250	(3.34)
Urinary incontinence	845	(1.40)	859	(1.74)	359	(2.07)	181	(2.42)
Phlebitis	754	(1.25)	744	(1.50)	356	(2.05)	244	(3.26)
Gout	3778	(6.25)	4129	(8.34)	1614	(9.31)	767	(10.24)
Gallbladder disease	2338	(3.87)	2063	(4.17)	736	(4.25)	338	(4.51)
Cataract	2681	(4.43)	2099	(4.24)	679	(3.92)	214	(2.86)
Anxiety	2893	(4.78)	2390	(4.83)	755	(4.36)	339	(4.52)
Erectile disfunction	953	(2.79)	760	(2.89)	192	(2.45)	63	(2.16)

*OW* Overweight, *OB-I* obesity class I, *OB-II* Obesity class II, *OB-III* obesity class II, *CVD* Cardiovascular disease, *T2D* type-2 diabetes, *CKD* chronic kidney disease, *MASLD* metabolic dysfunction-associated steatotic liver diseas, *GERD* gastroesophageal reflux disease, *OSA* obstructive sleep apnea

For all drugs considered, except cardiac preparations, lipid modifying agents, and hormones, the proportion of individuals with drug prescriptions increased according to increasing BMI during follow-up. The greatest differences were observed for antihypertensives, with proportions ranging from 55.7% for severe OW to 65.2% for OB-III, and antidiabetics, with individuals with prescriptions representing 19.1% of people with severe OW and 29.9% of individuals with OB-III. No particular trends emerged in terms of percentages of subjects with each laboratory test prescriptions during follow-up and BMI group, except for gamma-glutamyl transferase, aspartate aminotransferase, glycated hemoglobin, and basal insulin, for which proportions of people with ≥ 1 laboratory requests increased with BMI. In particular, subjects with requests for glycated hemoglobin ranged from 29.2% for severe OW to 43.7% for OB-III. Hospitalization rate also increased with BMI reaching 13.1% for OB-III (Table 2). Echocardiography, knee x-rays, spirometry, and polysomnogram were slightly more prescribed among people with higher BMI, while the opposite occurred for ergometric test, supra-aortic trunks echo-color doppler, and abdominal ultrasounds. Increasing proportions of subjects with referrals were observed in the OB-III group compared to those in the severe OW group for consultations with dietician/nutritionist (1.5% and 8.3%), endocrinologist (4.0% and 7.6%), diabetologist (8.2% and 13.2%), pulmonologist (4.2% and 9.4%), and psychiatrist/psychologist (1.7% and 4.2%) (Supplementary Information, Table 1S).

**Table 2:** Proportions of subjects with 1 + referral for each drug and laboratory test and 1 + hospitalization during follow-up stratified by body mass index (BMI) group

	Severe OW (*N* = 60,468)		OB-I (*N* = 49,486)		OB-II (*N* = 17,330)		OB-III (*N* = 7492)	
Intervention	*N*	(%)	*N*	(%)	*N*	(%)	*N*	(%)
*Drugs*								
Antihypertensives	33,683	(55.70)	30,980	(62.60)	11,314	(65.29)	4882	(65.16)
Cardiac drugs	3172	(5.25)	2697	(5.45)	930	(5.37)	373	(4.98)
Antithrombotics	17,369	(28.72)	15,308	(30.93)	5247	(30.28)	2342	(31.26)
Antidiabetics	11,574	(19.14)	11,786	(23.82)	4609	(26.60)	2238	(29.87)
Lipid modifying agents	18,514	(30.62)	16,118	(32.57)	5192	(29.96)	1968	(26.27)
Respiratory drugs	7481	(12.37)	6863	(13.87)	2749	(15.86)	1293	(17.26)
NSAIDs	18,484	(30.57)	16,440	(33.22)	6266	(36.16)	2806	(37.45)
Opioids	6141	(10.16)	6177	(12.48)	2396	(13.83)	1159	(15.47)
Antidepressants	7216	(11.93)	6051	(12.23)	2214	(12.78)	966	(12.89)
Antiacids	21,606	(35.73)	18,110	(36.60)	6374	(36.78)	2768	(36.95)
Thyroid therapies	4748	(7.85)	4537	(9.17)	1862	(10.74)	974	(13.00)
Hormones	564	(2.15)	421	(1.82)	158	(1.67)	63	(1.38)
*Laboratory tests*								
Fasting blood glucose	39,222	(64.86)	33,617	(67.93)	11,651	(67.23)	5127	(68.43)
Total cholesterol	37,465	(61.96)	31,971	(64.61)	11,118	(64.15)	4813	(64.24)
HDL cholesterol	36,265	(59.97)	30,978	(62.60)	10,717	(61.84)	4661	(62.21)
LDL cholesterol	30,626	(50.65)	25,730	(51.99)	8740	(50.43)	3695	(49.32)
Triglycerides	37,070	(61.31)	31,681	(64.02)	11,032	(63.66)	4783	(63.84)
GGT	20,362	(33.67)	17,734	(35.84)	6279	(36.23)	2764	(36.89)
AST	28,542	(47.20)	24,358	(49.22)	8606	(49.66)	3869	(51.64)
ALT	28,838	(47.69)	24,591	(49.69)	8512	(49.12)	3658	(48.83)
Creatinine	39,003	(64.50)	33,122	(66.93)	11,471	(66.19)	4990	(66.60)
Albumin	6719	(11.11)	5395	(10.90)	1864	(10.76)	855	(11.41)
CRP	8861	(14.65)	7356	(14.86)	2532	(14.61)	1098	(14.66)
Ferritin	7739	(12.80)	6219	(12.57)	2312	(13.34)	1076	(14.36)
TSH	17,621	(29.14)	15,408	(31.14)	5958	(34.38)	2873	(38.35)
Glycated hemoglobin	17,645	(29.18)	17,411	(35.18)	6786	(39.16)	3273	(43.69)
Basal insulin	631	(1.04)	798	(1.61)	483	(2.79)	383	(5.11)
*Hospitalizations*	6115	(10.11)	5464	(11.04)	2069	(11.94)	979	(13.07)

*OW* overweight, *OB-I* obesity class I, *OB-II* obesity class II, *OB-III* obesity class III, *NSAIDs* non-steroidal anti-inflammatory drugs, *HDL* high-density lipoprotein, *LDL* low-density lipoprotein, *GGT* gamma-glutamyl transferase, *AST* aspartate aminotransferase, *ALT* alanine aminotransferase, *CRP* C-reactive protein, *TSH* thyroid-stimulating hormone

Table 3 shows that the likelihood of being affected by nearly all the comorbidities considered during follow-up was significantly higher among individuals with obesity compared to severe OW. Exceptions included hypercholesterolemia and anxiety, for which individuals with obesity exhibited a lower risk, as well as gynecologic abnormalities, cataract, and erectile dysfunction, which showed no significant association with BMI. Moreover, for most conditions where individuals with obesity had a higher likelihood of being affected, the risk increased progressively with BMI. In particular, individuals in OB-III group had more than twice the odds of experiencing hypertension (OR 2.1), T2D (OR 2.5), knee osteoarthritis (OR 2.4), urinary incontinence (OR 2.2), phlebitis (OR 3.1), and gout (OR 2.9). Notably, the odds of presenting with OSA was more than six times higher (OR 6.9) (all *p* values < 0.001).

**Table 3:** Age- and sex-adjusted multivariate logistic models estimating the likelihood of presenting each comorbidity during follow-up

	OB-I (*N* = 49,486)		OB-II (*N* = 17,330)		OB-III (*N* = 7492)	
Comorbidity	OR	[95% CI]	OR	[95% CI]	OR	[95% CI]
T2D	1.38	[1.34 – 1.42]^*^	1.81	[1.74 – 1.89]^*^	2.49	[2.35 – 2.63]^*^
CVD	1.13	[1.10 – 1.16]^*^	1.20	[1.15 – 1.25]^*^	1.36	[1.28 – 1.45]^*^
Hypertension	1.39	[1.36 – 1.43]^*^	1.79	[1.72 – 1.86]^*^	2.12	[2.02 – 2.23]^*^
CKD	1.20	[1.12 – 1.28]^*^	1.50	[1.36 – 1.65]^*^	1.93	[1.68 – 2.22]^*^
Hypercholesterolemia	0.94	[0.91 – 0.97]^*^	0.78	[0.74 – 0.82]^*^	0.61	[0.56 – 0.67]^*^
Hypertriglyceridemia	1.29	[1.15 – 1.44]^*^	1.15	[0.98 – 1.36]	1.30	[1.03 – 1.64]^*^
MASLD	1.16	[1.10 – 1.24]^*^	1.29	[1.18 – 1.40]^*^	1.13	[0.99 – 1.28]
GERD	1.00	[0.97 – 1.03]	1.06	[1.01 – 1.11]^*^	1.08	[1.01 – 1.16]^*^
Gallbladder disease	1.08	[1.02 – 1.15]^*^	1.10	[1.02 – 1.21]^*^	1.22	[1.09 – 1.38]^*^
Asthma	1.13	[1.08 – 1.19]^*^	1.24	[1.16 – 1.32]^*\^	1.44	[1.32 – 1.56]^*^
OSA	1.93	[1.76 – 2.11]^*^	3.77	[3.40 – 4.18]^*^	6.92	[6.16 – 7.78]^*^
Depression	1.04	[0.98 – 1.11]	1.11	[1.02 – 1.21]^*^	1.27	[1.13 – 1.42]^*^
Anxiety	1.00	[0.94 – 1.05]	0.85	[0.78 – 0.92]^*^	0.85	[0.76 – 0.96]^*^
Knee osteoarthritis	1.48	[1.39 – 1.58]^*^	1.88	[1.73 – 2.04]^*^	2.44	[2.19 – 2.72]^*^
Back pain	1.05	[1.01 – 1.10]^*^	1.05	[1.00 – 1.11]	1.07	[0.99 – 1.16]
Phlebitis	1.22	[1.10 – 1.35]^*^	1.74	[1.53 – 1.98]^*^	3.07	[2.64 – 3.56]^*^
Gout	1.49	[1.42 – 1.56]^*^	2.05	[1.93 – 2.19]^*^	2.93	[2.68 – 3.19]^*^
Psoriasis	1.07	[1.00 – 1.16]	1.21	[1.09 – 1.34]^*^	1.45	[1.27 – 1.67]^*^
Cataract	1.01	[0.95 – 1.08]	1.08	[0.99 – 1.18]	0.97	[0.84 – 1.13]
Urinary incontinence	1.27	[1.15 – 1.40]^*^	1.59	[1.40 – 1.81]^*^	2.16	[1.83 – 2.55]^*^
Gynecologic abnormalities	1.10	[0.94 – 1.29]	1.04	[0.85 – 1.27]	1.23	[0.97 – 1.56]
Erectile dysfunction	1.05	[0.95 – 1.15]	0.91	[0.78 – 1.07]	0.85	[0.65 – 1.10]

The group of subjects with severe overweight (OW) was set as reference

*OB-I* obesity class I, *OB-II* obesity class II, *OB-III *obesity class III, *OR* Odds ratio,* CI* confidence interval, *T2D* type-2 diabetes, *CVD* cardiovascular disease, *CKD* chronic kidney disease, *MASLD* metabolic dysfunction-associated steatotic liver disease, *GERD* gastroesophageal reflux disease, *OSA* obstructive sleep apnea

^*^p value < 0.05

A dose–response relationship was observed also across most of the clinical interventions considered and for hospitalizations, with increasing ORs from OB-I to OB-III group (Supplementary Information, Table 2S).

## Discussion

The Italian Real-world overweight/Obesity Study (ITROS) is the first attempt to generate Italian real-word data on clinical characteristics and healthcare resources utilization (HRU) in people living with severe overweight or obesity, evaluating the management of the condition and its complications over time from a GP perspective. Major findings of the study underscore that overweight and obesity represent a burden for individuals, causing the worsening of general health status and an increase in HRUs over a period of just 12 months, and by the detection of a low grade of interventions and/or prescriptions specifically tailored for the management of overweight/obesity itself. These findings suggest a potential gap in obesity management at the GPs level, as patients may not be receiving the full spectrum of care needed.

The present study found that among individuals with obesity, 67% had class I, 23% had class II, and 10% had class III, which aligns with both European and local scientific literature. Indeed, a study conducted in the United Kingdom by Pearson-Stuttard [15] found these proportions to be 69%, 20%, and 11%. Similarly, an Italian study by Squadrito [16] reported that subjects with obesity class I, II, and III represented 65%, 23%, and 12%, respectively of people with BMI ≥ 30 kg/m^2^. A similar pattern was observed for demographic characteristics, with Pearson-Stuttard [15] and Squadrito [16] reporting a higher prevalence of women among subjects with obesity, and Squadrito [16] and Atella [17] reporting people with severe obesity being younger, consistently with findings from this study. Coherently with a study by Tsai, estimating that 8% only of primary care visits involve the management of overweight and obesity [18], few subjects had an additional BMI value recorded during follow-up. However, BMI proved to be a useful tool for assessing obesity-related characteristics in this study. Therefore, the use of this indicator should be promoted among healthcare providers.

All the evaluated clinical conditions were more frequently reported during follow-up across all BMI groups. This is indicative of the burden that severe overweight and obesity represent for the individual, causing the worsening of general health status even in a short period. The increased risk of several chronic diseases posed by obesity has been already reported by WHO [19] and the high prevalence of diseases such as hypertension, CVD, and T2D found in our cohort despite the relatively low overall mean age, represents a further confirmation. OSA, MASLD, and gallbladder disease showed the highest increase in terms of individuals affected when comparing follow-up and baseline data. Moreover, the likelihood of suffering of most of the clinical conditions we investigated rose with higher BMI. This finding aligns with Pearson-Stuttard [15], who reported a higher prevalence of obesity-related complications in individuals with elevated BMI and emphasized the need for effective weight-loss interventions. Notably, among people with higher BMI we also found increased proportions of individuals affected by age-related conditions like T2D and hypertension, with this potentially suggesting that the protective effect of younger age might not be sufficient to prevent people living with severe obesity from developing such conditions. All the clinical interventions implemented by GPs (prescriptions for drug treatments, referrals for laboratory tests, instrumental examinations, specialist visits) and hospitalizations were more frequent during follow-up across all BMI groups, likely reflecting the worsening of health conditions. This trend also highlights the persistent and escalating burden of obesity on healthcare systems. The increase in terms of HRU underscores the chronic nature of obesity and its associated comorbidities, necessitating continuous and comprehensive management. Moreover, we found that for most of the resources considered, people with severe obesity required more co-treatments, specialist visits, examinations, and hospitalizations. Among most frequently prescribed laboratory tests there were those concerning dyslipidemia and liver function, highlighting growing attention to these conditions. The increased demand for healthcare services among individuals with severe BMI underscores the importance of early intervention and continuous management to mitigate the long-term health and economic impacts of obesity. Notably, despite the increasing trend of most of the HRUs according to BMI class, the utilization of specialist services and specific diagnostic tests was limited. Also, prescription rates for obesity-related treatments were extremely low, with this probably reflecting the poor effectiveness of pharmacological treatments that were available at the time. In light of this, findings on hospitalizations from this study are even more relevant, as the high rate of hospitalizations observed may indicate that management and preventive interventions currently implemented are not yet adequate. A recent study by Artime and colleagues highlighted a gap in the clinical management of people living with overweight and obesity. The proportion of individuals with a formal diagnosis of overweight or obesity recorded in healthcare databases was notably low compared to the number of people actually identified as being affected by overweight and obesity. Furthermore, the proportion of individuals with documented BMI values within active healthcare populations was also limited. The authors of the study hypothesized that this under-reporting may be partly due to the insufficient recognition of obesity as a chronic disease [20]. GPs should play a crucial role in managing overweight and obesity, as they are typically the first healthcare providers patients consult. However, ITROS revealed that they seldom provide dietary or physical activity advice. It is instead fundamental they provide information to people living with overweight or obesity on their increased health risk and provide consistent advice on lifestyle modifications. Indeed a recent survey showed that receiving weight loss advice from a GP is strongly associated with a greater likelihood of trying to lose weight [21].

### Strength and limits

ITROS includes individuals registered on physician lists and not only those who demand health services, a key feature that reduces selection bias based on health status [10] and make results more generalizable to the Italian general population. According to authors’ knowledge, this is the first study providing such an extensive characterization of a very large sample of people living with obesity. Being so, ITROS contributes to the understanding of obesity in Italy and provides a foundation for future research and policy development aimed at tackling this growing public health issue. On the other hand, this analysis presents some limitations. First, we found high percentages of subjects without additional BMI measurements. This makes it impossible to determine the actual proportion of subjects who had significant variations in terms of BMI throughout the study. However, it is worth mentioning that this might be partially attributable to the ascertainment bias, with individuals without variations in terms of weight being less likely to have new assessments. Moreover, because a previous study by Atella dealing with the same data source estimated a probability of switching between BMI classes over time of 5% [10], and because the time-window observed for this study spanned for a relatively short period, it is reasonable assuming that our findings are not significantly affected by potential BMI class switches. Second, few data were found pertaining to obesity treatments, which might be attributed to the limited pharmacological armamentarium available at the time. This underscores the need for future research utilizing more up-to-date data to better engage GPs in this critical aspect of obesity management. Third, information on bariatric surgery were recorded for a very small number of individuals, with this potentially reflecting a limit of the database, which is not able to track patients when following therapeutical pathways falling outside GP’s domain, like for example that represented by obesity centers. However, the focus of this study was on primary care. Fourth, it should be considered that IQVIA LPD estimates on interventions occurring in the private sector might be under-estimated as in some cases a referral might not be needed. Fifth, IQVIA LPD collects information on drugs prescriptions and referrals for examination or specialist visits, thus the data does not ensure that the patient consumed the treatment or take the exam/visit. However, it should be considered that the aim of the study was the taking charge and management by GPs of patients living with severe overweight and obesity, rather than the patient healthcare pathway. Sixth, the relatively short observation period represents a limitation of the present study. Future research incorporating longer follow-up durations may provide deeper insights into the complex and evolving interplay between obesity, its associated complications, HRU, and their trajectories over time. Finally, an unexpected decrease in the risk of hypercholesterolemia was observed with increasing BMI. This finding is consistent with the absence of a clear association between the degree of obesity and the frequency of lipid-lowering agent prescriptions observed. It is therefore plausible that, while the overall number of individuals treated with lipid-lowering agents remains stable across BMI categories, the proportion of those receiving statins may increase with higher BMI, whereas the use of agents not specifically targeting cholesterol may decrease—resulting in a stable overall proportion of “lipid-lowering drug” users across BMI groups. Unfortunately, the sample size does not allow for sub-analyses by specific drug categories, and this limitation should be acknowledged as a constraint of the study. Nevertheless, this topic remains of particular interest for understanding the cardiovascular risk associated with obesity and warrants further investigation in larger cohorts.

### What is already known on this subject?

Regardless of programs implemented by the Italian Government [1], recent reports indicate that in the last 20 years people living with overweight and obesity had an increase in prevalence of 14% and 38% respectively [8]. A real-world evidence study was needed to understand the state of the art of obesity management in primary care in light of the key role GPs might play in the Italian healthcare system.

### What this study adds?

Severe overweight and obesity represent a burden for the individual causing the worsening of general health status and an increase in HRUs over a period of just 12 months. Moreover, the likelihood of suffering of most of the clinical conditions and the need for clinical interventions investigated increased with increasing BMI.

## Conclusion

Our findings suggest a potential gap in obesity management as patients may not be receiving the full spectrum of care needed. Public health interventions might focus on supporting GPs through training programs and resources emphasizing the importance of obesity management. By improving GP awareness and equipping them with clinical tools and evidence-based knowledge, the management of obesity and its complications can be enhanced, ultimately improving patient outcomes and reducing the healthcare burden.

## Supplementary Information

Below is the link to the electronic supplementary material.

Supplementary Material 1.

## Data Availability

Data supporting findings of this work are available from IQVIA, but restrictions apply to their availability. Data were used under license for the current work and are not publicly available. However, they are available from the corresponding author upon reasonable request and with the permission of IQVIA.
