# Supplementary material for: The burden of obesity in primary care in Italy: Italian real-world overweight/obesity study (ITROS)
Source: Eat Weight Disord. 2025 Oct 22;30(1):83. doi: 10.1007/s40519-025-01791-8 (PMC12545663; doi:10.1007/s40519-025-01791-8)
Supplement: Supplementary file 1 — Supplementary Material 1. [file 40519_2025_1791_MOESM1_ESM.docx]

**Supplementary Information**

**Figure 1S. Patients Flow Diagram**

**
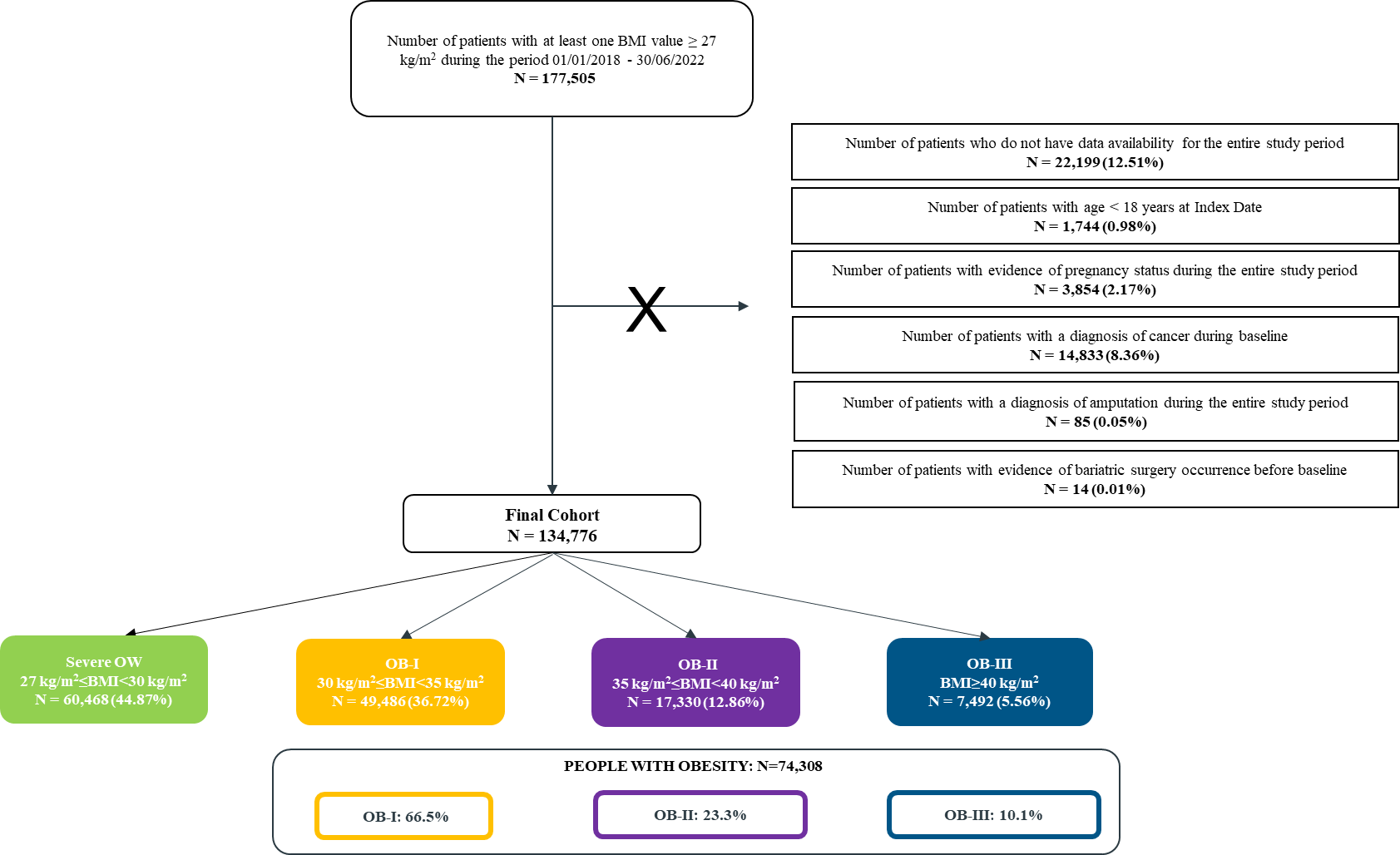
**

BMI: body mass index. OW: overweight. OB-I: obesity class I. OB-II: obesity class II. OB-III: obesity class III.

**Table 1S. Proportions of subjects with 1+ referral for each instrumental examination and specialist visit during follow-up stratified by body mass index (BMI) group**

|  | **Severe OW**  **(N=60,468)** | | **OB-I**  **(N=49,486)** | | **OB-II**  **(N=17,330)** | | **OB-III**  **(N=7,492)** | |
| --- | --- | --- | --- | --- | --- | --- | --- | --- |
| **Intervention** | **N** | **(%)** | **N** | **(%)** | **N** | **(%)** | **N** | **(%)** |
| *Instrumental Examinations* | |  |  |  |  |  |  |  |
| ECG | 15,746 | (26.04) | 13,784 | (27.85) | 4,789 | (27.63) | 2,043 | (27.27) |
| Echocardiography | 2,194 | (3.63) | 2,017 | (4.08) | 716 | (4.13) | 329 | (4.39) |
| Ergometric test | 1,836 | (3.04) | 1,425 | (2.88) | 400 | (2.31) | 115 | (1.53) |
| Supra-aortic trunks echo-color doppler | 6,751 | (11.16) | 5,497 | (11.11) | 1,760 | (10.16) | 664 | (8.86) |
| Abdominal ultrasounds | 8,891 | (14.70) | 7,084 | (14.32) | 2,403 | (13.87) | 1,010 | (13.48) |
| Abdominal CT | 1,013 | (1.68) | 830 | (1.68) | 291 | (1.68) | 128 | (1.71) |
| Chest x-rays | 3,940 | (6.52) | 3,379 | (6.83) | 1,093 | (6.31) | 475 | (6.34) |
| Chest CT | 994 | (1.64) | 834 | (1.69) | 290 | (1.67) | 114 | (1.52) |
| Knee x-rays | 2,188 | (3.62) | 2,310 | (4.67) | 966 | (5.57) | 439 | (5.86) |
| Knee MRI | 956 | (1.58) | 937 | (1.89) | 335 | (1.93) | 134 | (1.79) |
| Spine x-rays | 4,034 | (6.67) | 3,594 | (7.26) | 1,250 | (7.21) | 550 | (7.34) |
| Spine MRI | 2,422 | (4.01) | 2,062 | (4.17) | 750 | (4.33) | 243 | (3.24) |
| Spirometry | 2,573 | (4.26) | 2,329 | (4.71) | 914 | (5.27) | 439 | (5.86) |
| Polysomnogram | 398 | (0.66) | 561 | (1.13) | 330 | (1.90) | 262 | (3.50) |
| Esophagogastroduodenoscopy | 1,729 | (2.86) | 1,312 | (2.65) | 449 | (2.59) | 234 | (3.12) |
| Transvaginal ultrasound | 759 | (2.89) | 680 | (2.94) | 274 | (2.89) | 142 | (3.10) |
| Thyroid ultrasound | 2,457 | (4.06) | 2,006 | (4.05) | 713 | (4.11) | 336 | (4.48) |
| Hip x-rays | 1,452 | (2.40) | 1,272 | (2.57) | 460 | (2.65) | 190 | (2.54) |
| *Specialist visits* |  |  |  |  |  |  |  |  |
| Dietician/nutritionist | 926 | (1.54) | 1,544 | (3.12) | 888 | (5.12) | 619 | (8.27) |
| Endocrinologist | 2,402 | (3.97) | 2,215 | (4.47) | 1,020 | (5.88) | 570 | (7.61) |
| Internal medicine doctor | 680 | (1.12) | 549 | (1.11) | 241 | (1.39) | 132 | (1.76) |
| Cardiologist | 12,485 | (20.65) | 11,019 | (22.27) | 3,895 | (22.48) | 1,658 | (22.13) |
| Diabetologist | 4,982 | (8.24) | 4,995 | (10.09) | 2,028 | (11.70) | 990 | (13.21) |
| Pulmonologist | 2,563 | (4.24) | 2,568 | (5.19) | 1,179 | (6.81) | 701 | (9.36) |
| Orthopedist | 5,885 | (9.73) | 5,486 | (11.09) | 1,972 | (11.38) | 835 | (11.14) |
| Psychiatrist/psychologist | 1,036 | (1.71) | 1,038 | (2.09) | 486 | (2.80) | 316 | (4.22) |
| Dermatologist | 4,460 | (7.38) | 3,485 | (7.04) | 1,188 | (6.85) | 496 | (6.63) |
| Gynecologist | 1,757 | (6.68) | 1,472 | (6.36) | 628 | (6.62) | 290 | (6.33) |
| Nephrologist | 1,144 | (1.89) | 1,027 | (2.07) | 405 | (2.34) | 165 | (2.20) |
| Physiatrist | 3,473 | (5.74) | 3,065 | (6.19) | 1,053 | (6.08) | 452 | (6.04) |
| Urologist | 3,677 | (6.08) | 2,676 | (5.41) | 765 | (4.42) | 269 | (3.59) |
| Gastroenterologist | 1,336 | (2.21) | 963 | (1.94) | 312 | (1.80) | 146 | (1.94) |
| Neurosurgeon | 729 | (1.20) | 680 | (1.38) | 217 | (1.25) | 73 | (0.97) |

BMI: body mass index. OW: overweight. OB-I: obesity class I. OB-II: obesity class II. OB-III: obesity class III. ECG: electrocardiogram. CT: computed tomography. MRI: magnetic resonance imaging.

**Table 2S. Age- and sex-adjusted multivariate logistic models estimating the likelihood of presenting 1+ referral for each intervention by general practitioner (GP) and 1+ hospitalization during follow-up. The group of subjects with severe overweight (OW) was set as reference**

|  | OB-I (N=49,486) | | OB-II (N=17,330) | | OB-III (N=7,492) | |
| --- | --- | --- | --- | --- | --- | --- |
| Intervention | **OR** | **[95% CI]** | **OR** | **[95% CI]** | **OR** | **[95% CI]** |
| *Drugs* |  |  |  |  |  |  |
| Antihypertensives | 1.56 | [1.51 – 1.60]^*^ | 2.21 | [2.12 – 2.30]^*^ | 2.99 | [2.82 – 3.17]^*^ |
| Cardiac drugs | 1.12 | [1.06 – 1.19]^*^ | 1.33 | [1.23 – 1.44]^*^ | 1.58 | [1.41 – 1.78]^*^ |
| Antithrombotics | 1.23 | [1.19 – 1.27]^*^ | 1.45 | [1.39 – 1.52]^*^ | 2.09 | [1.97 – 2.22]^*^ |
| Antidiabetics | 1.41 | [1.37 – 1.45]^*^ | 1.87 | [1.80 – 1.95]^*^ | 2.67 | [2.52 – 2.83]^*^ |
| Lipid modifying agents | 1.16 | [1.13 – 1.19]^*^ | 1.15 | [1.11 – 1.20]^*^ | 1.15 | [1.08 – 1.21]^*^ |
| Respiratory drugs | 1.15 | [1.11 – 1.19]^*^ | 1.38 | [1.32 – 1.45]^*^ | 1.60 | [1.50 – 1.71]^*^ |
| NSAIDs | 1.12 | [1.09 – 1.15]^*^ | 1.26 | [1.22 – 1.31]^*^ | 1.35 | [1.28 – 1.41]^*^ |
| Opioids | 1.28 | [1.23 – 1.33]^*^ | 1.47 | [1.40 – 1.55]^*^ | 1.82 | [1.70 – 1.96]^*^ |
| Antidepressants | 1.01 | [0.97 – 1.05] | 1.03 | [0.98 – 1.09] | 1.06 | [0.99 – 1.14] |
| Antiacids | 1.06 | [1.03 – 1.08]^*^ | 1.13 | [1.08 – 1.17]^*^ | 1.27 | [1.20 – 1.34]^*^ |
| Thyroid therapies | 1.14 | [1.09 – 1.19]^*^ | 1.25 | [1.18 – 1.33]^*^ | 1.50 | [1.39 – 1.62]^*^ |
| Hormones | 0.84 | [0.74 – 0.95]^*^ | 0.75 | [0.62 – 0.89]^*^ | 0.57 | [0.44 – 0.74]^*^ |
| *Laboratory tests* |  |  |  |  |  |  |
| Fasting blood glucose | 1.17 | [1.14 – 1.20]^*^ | 1.19 | [1.15 – 1.23]^*^ | 1.37 | [1.30 – 1.44]^*^ |
| Total cholesterol | 1.14 | [1.11 – 1.16]^*^ | 1.16 | [1.12 – 1.21]^*^ | 1.25 | [1.19 – 1.32]^*^ |
| HDL cholesterol | 1.13 | [1.11 – 1.16]^*^ | 1.15 | [1.11 – 1.19]^*^ | 1.26 | [1.19 – 1.32]^*^ |
| LDL cholesterol | 1.07 | [1.05 – 1.10]^*^ | 1.06 | [1.02 – 1.10]^*^ | 1.10 | [1.05 – 1.16]^*^ |
| Triglycerides | 1.14 | [1.11 – 1.17]^*^ | 1.17 | [1.13 – 1.22]^*^ | 1.27 | [1.21 – 1.34]^*^ |
| GGT | 1.11 | [1.08 – 1.14]^*^ | 1.16 | [1.11 – 1.20]^*^ | 1.23 | [1.17 – 1.29]^*^ |
| AST | 1.09 | [1.07 – 1.12]^*^ | 1.15 | [1.11 – 1.19]^*^ | 1.30 | [1.24 – 1.37]^*^ |
| ALT | 1.09 | [1.07 – 1.12]^*^ | 1.10 | [1.06 – 1.13]^*^ | 1.13 | [1.07 – 1.18]^*^ |
| Creatinine | 1.14 | 1.11 – 1.16]^*^ | 1.16 | [1.12 – 1.21]^*^ | 1.31 | [1.25 – 1.38]^*^ |
| Albumin | 0.98 | [0.95 – 1.02] | 1.00 | [0.94 – 1.05] | 1.12 | [1.04 – 1.21]^*^ |
| CRP | 1.01 | [0.98 – 1.04] | 0.98 | [0.93 – 1.03] | 0.99 | [0.93 – 1.06] |
| Ferritin | 0.96 | [0.93 – 1.00]^*^ | 0.99 | [0.95 – 1.05] | 1.06 | [0.99 – 1.14] |
| TSH | 1.07 | [1.04 – 1.10]^*^ | 1.15 | [1.11 – 1.20]^*^ | 1.29 | [1.22 – 1.36]^*^ |
| Glycated hemoglobin | 1.39 | [1.35 – 1.42]^*^ | 1.84 | [1.77 – 1.91]^*^ | 2.59 | [2.46 – 2.73]^*^ |
| Basal insulin | 1.52 | [1.37 – 1.69]^*^ | 2.38 | [2.11 – 2.67]^*^ | 3.83 | [3.36 – 4.37]^*^ |
| *Instrumental examinations* |  |  |  |  |  |  |
| ECG | 1.12 | [1.09 – 1.15]^*^ | 1.18 | [1.14 – 1.23]^*^ | 1.27 | [1.20 – 1.34]^*^ |
| Echocardiography | 1.15 | [1.08 – 1.23]^*^ | 1.24 | [1.14 – 1.35]^*^ | 1.44 | [1.28 – 1.63]^*^ |
| Ergometric test | 0.98 | [0.91 – 1.05] | 0.84 | [0.75 – 0.94]^*^ | 0.60 | [0.50 – 0.73]^*^ |
| Supra-aortic trunks echo-color doppler | 1.02 | [0.98 – 1.06] | 1.01 | [0.95 – 1.06] | 0.98 | [0.90 – 1.06] |
| Abdominal ultrasounds | 0.98 | [0.94 – 1.01] | 0.97 | [0.92 – 1.01] | 0.97 | [0.90 – 1.04] |
| Abdominal CT | 1.03 | [0.93 – 1.12] | 1.10 | [0.96 – 1.25] | 1.23 | [1.02 – 1.48]^*^ |
| Chest x-rays | 1.07 | [1.02 – 1.12]^*^ | 1.03 | [0.96 – 1.11] | 1.12 | [1.01 – 1.24]^*^ |
| Chest CT | 1.06 | [0.97 – 1.17] | 1.16 | [1.02 – 1.33]^*^ | 1.20 | [0.98 – 1.46] |
| Knee x-rays | 1.31 | [1.23 – 1.39]^*^ | 1.60 | [1.48 – 1.73]^*^ | 1.78 | [1.60 – 1.98]^*^ |
| Knee MRI | 1.20 | [1.09 – 1.31]^*^ | 1.20 | [1.06 – 1.36]^*^ | 1.08 | [0.89 – 1.29] |
| Spine x-rays | 1.08 | [1.04 – 1.14]^*^ | 1.06 | [0.99 – 1.13] | 1.08 | [0.98 – 1.18] |
| Spine MRI | 1.03 | [0.97 – 1.10] | 1.05 | [0.97 – 1.15] | 0.76 | [0.66 – 0.87]^*^ |
| Spirometry | 1.13 | [1.07 – 1.20]^*^ | 1.34 | [1.24 – 1.45]^*^ | 1.59 | [1.44 – 1.77]^*^ |
| Polysomnogram | 1.78 | [1.57 – 2.03]^*^ | 3.24 | [2.80 – 3.76]^*^ | 6.47 | [5.51 – 7.60]^*^ |
| Esophagogastroduodenoscopy | 0.92 | [0.85 – 0.99]^*^ | 0.88 | [0.79 – 0.98]^*^ | 1.05 | [0.92 – 1.21] |
| Transvaginal ultrasound1 | 1.01 | [0.91 – 1.12] | 0.96 | [0.83 – 1.10] | 0.95 | [0.79 – 1.14] |
| Thyroid ultrasound | 0.96 | [0.90 – 1.02] | 0.89 | [0.81 – 0.97]^*^ | 0.90 | [0.80 – 1.01] |
| Hip x-rays | 1.07 | [1.00 – 1.16] | 1.13 | [1.01 – 1.26]^*^ | 1.15 | [0.99 – 1.35] |
| *Specialist visits* |  |  |  |  |  |  |
| Dietician/nutritionist | 2.04 | [1.88 – 2.21]^*^ | 3.22 | [2.93 – 3.53]^*^ | 4.96 | [4.46 – 5.51]^*^ |
| Endocrinologist | 1.09 | [0.03 – 1.16]^*^ | 1.33 | [1.24 – 1.44]^*^ | 1.62 | [1.47 – 1.78]^*^ |
| Internal medicine doctor | 0.99 | [0.89 – 1.11] | 1.27 | [1.10 – 1.48]^*^ | 1.67 | [1.38 – 2.02]^*^ |
| Cardiologist | 1.14 | [1.10 – 1.17]^*^ | 1.25 | [1.20 – 1.30]^*^ | 1.38 | [1.30 – 1.47]^*^ |
| Diabetologist | 1.30 | [1.25 – 1.36]^*^ | 1.70 | [1.61 – 1.80]^*^ | 2.25 | [2.08 – 2.42]^*^ |
| Pulmonologist | 1.27 | [1.20 – 1.34]^*^ | 1.83 | [1.70 – 1.96]^*^ | 2.85 | [2.61 – 3.11]^*^ |
| Orthopedist | 1.15 | [1.11 – 1.20]^*^ | 1.18 | [1.12 – 1.25]^*^ | 1.18 | [1.09 – 1.28]^*^ |
| Psychiatrist/psychologist | 1.21 | [1.10 – 1.31]^*^ | 1.51 | [1.36 – 1.69]^*^ | 2.11 | [1.85 – 2.40]^*^ |
| Dermatologist | 0.95 | [0.91 – 1.00]^*^ | 0.93 | [0.87 – 0.99]^*^ | 0.90 | [0.81 – 0.99]^*^ |
| Gynecologist^1^ | 0.94 | [0.88 – 1.01] | 0.95 | [0.86 – 1.04] | 0.83 | [0.73 – 0.94]^*^ |
| Nephrologist | 1.17 | [1.07 – 1.28]^*^ | 1.55 | [1.38 – 1.74]^*^ | 1.82 | [1.53 – 2.15]^*^ |
| Physiatrist | 1.08 | [1.02 – 1.13]^*^ | 1.04 | [0.97 – 1.12] | 1.06 | [0.96 – 1.18] |
| Urologist | 0.95 | [0.90 – 1.00]^*^ | 0.93 | [0.86 – 1.01] | 0.94 | [0.82 – 1.07] |
| Gastroenterologist | 0.87 | [0.80 – 0.95]^*^ | 0.80 | [0.70 – 0.90]^*^ | 0.85 | [0.72 – 1.01] |
| Neurosurgeon | 1.14 | [1.03 – 1.27]^*^ | 1.04 | [0.89 – 1.21] | 0.81 | [0.64 – 1.04] |
| *Hospitalizations* | 1.12 | [1.08 – 1.17]^*^ | 1.30 | [1.23 – 1.37]^*^ | 1.56 | [1.45 – 1.68]^*^ |

OB-I: obesity class I. OB-II: obesity class II. OB-III: obesity class III. OR: Odds ratio. CI: confidence interval. NSAIDs: non-steroidal anti-inflammatory drugs. HDL: high-density lipoprotein. LDL: low-density lipoprotein. GGT: gamma-glutamyl transferase. AST: aspartate aminotransferase. ALT: alanine aminotransferase. CRP: C-reactive protein. TSH: thyroid-stimulating hormone. ECG: electrocardiogram. CT: computed tomography. MRI: magnetic resonance imaging.

^*^ p-value<0.05
